# Systematic Review of Psychological Interventions for Quality of Life, Mental Health, and Hair Growth in Alopecia Areata and Scarring Alopecia

**DOI:** 10.3390/jcm12030964

**Published:** 2023-01-26

**Authors:** Jessica Maloh, Tess Engel, Nicole Natarelli, Yvonne Nong, Alina Zufall, Raja K. Sivamani

**Affiliations:** 1 Zen Dermatology, Sacramento, CA 95819, USA; 2 Integrative Skin Science and Research, Sacramento, CA 95815, USA; 3 School of Medicine, University of California-Davis, Sacramento, CA 95817, USA; 4 Morsani College of Medicine, University of South Florida, Tampa, FL 33602, USA; 5 Department of Dermatology, University of California-Davis, Sacramento, CA 95816, USA; 6 Department of Dermatology, New York Medical College, Valhalla, NY 10595, USA; 7 College of Medicine, California Northstate University, Elk Grove, CA 95757, USA; 8 Pacific Skin Institute, Sacramento, CA 95815, USA

**Keywords:** psychology, hair loss, alopecia areata, mental health

## Abstract

Alopecia is associated with significant psychological burden. There is limited evidence on the use of psychological interventions in conditions of hair loss. This manuscript systematically reviews the current state of literature on psychological treatments for quality of life, mental health, and hair growth in various forms of alopecia. PubMed and Embase were searched with predefined inclusion and exclusion criteria. Reference lists were also examined for relevant studies. Nine articles met our criteria and are included in this review. Eight of the articles related to alopecia areata and one related to scarring alopecia. Mindfulness-based stress reduction (MBSR) was found to improve quality of life-related subjective symptoms, relationship impacts, anxiety, phobia, distress, and psychological symptom intensity. Alopecia-specific collocated behavioral health (CLBH) treatment showed a trend for psychosocial improvement in areas such as appearance shame, activity avoidance, negative emotions, and coping. Hypnotherapy was found to improve anxiety and depression, quality of life measures, and alexithymia. There was also some evidence for significant hair growth with hypnosis, but the data are mixed. Psychotherapy combined with immunotherapy led to more hair growth, and supported self-confidence. Finally, coping strategies modulated the subjective burden of alopecia, and were associated with disease improvement. Further research will be necessary to better establish the efficacy and optimal administration of these interventions in alopecia.

## 1. Introduction

Alopecia is prevalent in both female and male populations and is associated with significant psychological burden [1]. Different forms of alopecia can result in losing hair either in patches, partially, diffusely, or completely [2]. Furthermore, some forms of alopecia are temporary or reversible, while others can be chronic or permanent [2]. Arriving at the appropriate diagnosis is important to set the stage for treatment and prognosis, but may not capture the profound emotional consequences and impediments to quality of life accompanying hair loss.

Patients with hair loss can experience feelings of sadness, shame, social isolation, and an increased risk of mental health disorders [3,4,5]. For example, one publication reports that relative to a healthy population, those with alopecia areata (AA) have a significantly greater prevalence of major depressive episodes, generalized anxiety disorder, social phobia, and paranoid disorder [5]. Similarly, children with AA have also been found to have more symptoms of anxiety and depression, and lower levels of self-esteem relative to control groups [6,7]. Furthermore, in androgenic alopecia (AGA) and primary cicatricial alopecia (PCA), there are significant reductions in quality of life, coupled with low perceived control over the hair loss and its treatment [8,9]. To best support patients, and optimize their overall wellbeing, clinicians must recognize and address these psychological consequences of alopecia in their treatment plans.

First-line treatments depend on the exact form of alopecia being treated and may include intralesional or topical steroids, oral finasteride, or topical minoxidil [10,11]. Steroids can address the underlying immune dysregulation and inflammation associated with hair loss conditions such as AA, while finasteride can serve as an antiandrogenic agent to address the hormonal pathology associated with AGA [12,13,14]. Topical minoxidil may also have antiandrogenic activity, may increase blood flow to the scalp, and is another treatment option for AGA [15,16]. While these interventions have evidence for inducing short-term hair growth, the benefits may not be maintained without continual use as hair loss can recur in the absence of treatment [10].

Alopecia can be difficult to manage, especially in scarring alopecia or more severe cases of AA, such as alopecia totalis and alopecia universalis, where there are lower chances of recovery [17]. In treatment-resistant patients without clinical improvement in hair loss, we must be prepared with resources and plans to support psychoemotional well-being. Additionally, even with effective treatments, it is important to keep in mind that complete hair regrowth can take time, during which psychological distress and low self-esteem can persist.

There is some research investigating the use of psychological interventions, such as mindfulness, hypnosis, and psychotherapy in individuals experiencing hair loss [18,19,20,21,22]. Overall, these studies suggest that psychological treatments can offer patients with alopecia improvements in depression, anxiety, quality of life, and self-confidence [18,20,21,23]. Moreover, some psychological interventions may not only support quality life and emotional wellness, but also, hair regrowth [24,25].

While this area of research is gaining traction, the current state of evidence is limited. Other reviews have discussed psychological interventions in alopecia, mainly psychotherapy, hypnosis, and mindfulness [26,27,28]. However, to our knowledge, this is the first review to focus on psychological interventions that also include behavioral therapy and coping strategies for patients with hair loss. The objective of this review is to summarize and discuss the literature surrounding mindfulness practices, behavioral therapy, hypnotherapy, psychotherapy, and coping strategies in supporting quality of life and clinical outcomes in patients with alopecia areata and scarring alopecia.

## 2. Methods

### 2.1. Search Strategy

A systematic search was performed on the PubMed and Embase databases for publications of original data on psychological interventions in alopecia. This systematic review was not registered and a protocol was not prepared. Specifically, we were interested in investigating the effects of psychological interventions on quality of life, mental health, and hair growth. In June 2022, we used the following search terms: alopecia, hypnosis, mindfulness, meditation, psychotherapy, quality of life, anxiety, mental health, and depression. Searches were not limited to any particular time frame. The reference lists of articles retrieved were also examined for additional relevant data.

### 2.2. Inclusion/Exclusion Criteria

Inclusion criteria consisted of (1) human studies; (2) original data specific to those with alopecia; (3) at least one psychological intervention with an outcome measure for either quality of life, mental health, or hair growth. Studies were excluded if they (1) were not in English; (2) examined hair loss caused by primary psychogenic disorders such as trichotillomania or by pharmaceutical interventions such as chemotherapy.

### 2.3. Data Collection and Search Results

**Figure 1:**
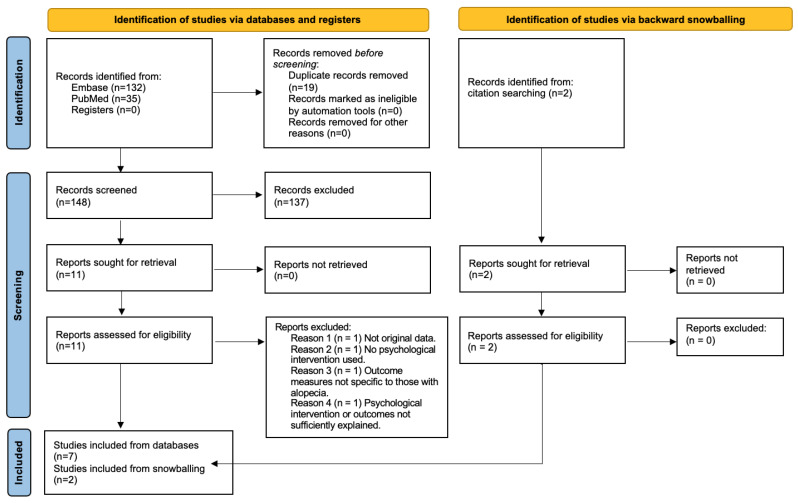
Flow chart of search strategy according to PRISMA.

Our database search yielded 167 results. Nineteen of these were duplicates and were removed. With the remaining 148 results, two authors independently reviewed the titles and abstract for relevance and any uncertain studies or discrepancies were discussed mutually with evaluation of the full text. From these articles, the outcomes of interest were quality of life, mental health status, and/or hair growth in a population with alopecia where at least one psychological tool was analyzed. Two articles were retrieved from citation searching via backward snowballing. A total of nine papers met the inclusion/exclusion criteria and the full-text versions were used to extract data and include in this review. The overall search strategy is outlined in Figure 1.

## 3. Results

### 3.1. Mindfulness

A prospective pilot study by Gallo et al. in 2017 investigated the effect of mindfulness-based practices on the quality of life of 16 patients with moderate to severe alopecia areata [18]. All 16 patients continued their usual AA treatments, while only 8 of the subjects concurrently participated in an eight-week Mindfulness-Based Stress Reduction (MBSR) program. This program aims to cultivate mindfulness through exercises such as yoga, body scans, and seated meditations during weekly in-person group sessions, and during daily application outside of scheduled class time [18].

Quality of life measures, self-reported psychological symptoms, and perceived levels of stress were assessed at baseline, at the end of the program, and at 6 months after the study [18]. These outcomes were assessed using the Alopecia Areata Quality of Life Index Questionnaire (AA-QLI), the Brief Symptom Inventory (BSI), and the Perceived Stress Scale (PSS), respectively.

The AA-QLI is a quality-of-life questionnaire specific to AA. Here, patients answer 21 questions with regards to how much AA has affected them over the last month in three different categories: relationships (ex: “I feel I have sexual difficulties because of alopecia areata”), subjective symptoms (ex: “I am sad about the appearance of my hair ⁄ eyebrows ⁄ eyelashes”), and objective signs (ex: “My scalp is visible”) [29]. The BSI consists of 53 questions that cover nine dimensions of psychological distress and psychological disorders [30]. These dimensions include somatization, obsession–compulsion, interpersonal sensitivity, depression, anxiety, hostility, phobic anxiety, paranoid ideation, and psychoticism [30]. The PSS consists of 14 items about stress-related thoughts and feelings over the course of the previous month [31]. These items include questions such, “In the last month, how often have you felt that you were unable to control the important things in your life?” [31].

Unlike the control group, patients who underwent the 2-month program had significant improvement in AA-QLI subjective symptoms and relationship impacts, as well as BSI anxiety and phobia [18]. No significant changes were found in the MBSR nor in the control group. However, after 6 months, improvements were maintained in AA-QLI relationships and BSI anxiety in the MBSR group relative to baseline [18]. Interestingly, the MBSR program was able to support quality of life and psychological wellness, even though there were no significant improvements in AA over the course of the study [18]. This suggests that mindfulness may be a useful strategy in coping with the distress associated with ongoing hair loss. While these findings are promising, it is important to note that this study had a small sample size and that the MBSR group was found to have worse baseline scores compared to control, which could have skewed the data and its interpretation [18].

Similarly, a case series investigated the effects of an eight-session mindfulness-based cognitive therapy (MBCT) intervention in five patients with AA [21]. One patient’s data were ultimately excluded due to missing data, although the remaining four patients experienced reductions in idiographic measures of social anxiety from baseline to follow up. Using questionnaires relating to trait mindfulness, social anxiety, depression, anxiety, and quality of life, the authors conclude that the magnitude of improvement in social anxiety is greater between baseline and follow-up than baseline and postintervention, highlighting the potential importance of MBCT treatment maintenance. This contrasts with the results presented by Gallo et al., who found MBSR-associated improvements in AA-QLI and BSI anxiety to be maintained 6 months postintervention [18]. Further investigation is required to assess the duration by which positive effects last following MBCT intervention. Interestingly, the authors of the MBCT case series discuss two participants who practiced MBCT-associated exercises frequently between sessions and depicted significant improvement in standardized measures of well-being from baseline to follow-up [21]. It is possible that the reduction in social anxiety and improvement in overall well-being from MBCT intervention is dependent on intervention frequency. Further investigation on mindfulness-based interventions with larger sample sizes is warranted. These results are summarized in Table 1.

### 3.2. Collocated Behavioral Health Treatment

A randomized, controlled pilot study to investigated the efficacy of AA-specific collocated behavioral health (CLBH) treatment on several measures of psychosocial functioning [22]. Twenty patients with AA received up to two 30-min sessions, whereas ten control patients did not receive psychotherapy. The CLBH group reported better psychosocial functioning trends in several areas including appearance shame (*p* = 0.18), activity avoidance (*p* = 0.17), negative emotions (*p* = 0.13), and coping (*p* = 0.12) compared to the control group. Overall, CLBH was rated as beneficial by treatment participants and 100% reported an increase in the satisfaction in their care. The authors specify that implementation of the CLBH treatment was highly feasible with no clinic disruption. The results of this study are promising, although they are limited by its small sample size [22]. Nevertheless, the promising results in a pilot study support further follow-up studies in a larger cohort.

### 3.3. Hypnotherapy

The earliest investigation on hypnotherapy for alopecia was conducted by Harrison and Stepanek in 1991 [32]. Five patients with refractory alopecia completed the treatment and evaluation; 3 patients had non-scarring alopecia and 2 patients had scarring alopecia. Participants underwent 10–12 forty-five-minute sessions of hypnotherapy over the course of 3 months which included techniques of direct and indirect suggestions and ego strengthening [32]. Patients were also given a recording to use regularly outside of the sessions. At baseline, all had some level of anxiety, but at the end of treatment, all reported a feeling of well-being. The measure used to assess well-being was not reported by the authors [32]. With regards to alopecia clinical outcomes, three patients experienced partial hair regrowth, one patient had significant regrowth, and one patient experienced no change in hair regrowth [32]. Interestingly, two of the individuals who experienced partial hair regrowth had scarring alopecia. This study suggests that hypnotherapy may benefit patients with both scarring and non-scarring hair loss, but further research will be necessary.

In 2006, Willemsen et al. investigated the use of hypnosis in a clinical trial for patients with AA [24]. Here, hypnotherapy was offered to establish a sense of relaxation in patients and to encourage symptom-oriented visualizations. For example, patients were asked to imagine the sensation of the warm sun on their scalp, to create a personal metaphor for growing hairs, and for those with social phobia or agoraphobia, to visualize a sense of emotional ease and greater self-esteem [24]. In total, 21 refractory hair loss patients completed the study; 9 of these patients had alopecia totalis or universalis variants of AA, and 12 had extensive AA [24]. In cases where other alopecia treatments were being used, hypnosis was offered adjunctly, and in cases where no other interventions were being utilized at the time of the study, hypnosis served as the standalone therapy. Hypnosis sessions were scheduled every 3 weeks. All subjects participated in at least three sessions and were instructed to practice self-hypnosis two times each week [24].

Psychological well-being was measured at baseline and at the end of treatment with the Symptom Check List (SCL) total score, which assesses psychoneuroticism. The SCL also assesses eight psychological symptoms (phobia, anxiety, depression, somatization, psychoticism, interpersonal sensitivity, hostility, and sleep problems), but of these, only anxiety and depression were analyzed in this study [24]. The investigators also assessed alopecia improvement by clinical examination and defined significant hair growth as 75–100% scalp growth [24]. Relative to baseline measures, there was significant improvement in the SCL total score, and anxiety and depression subscales with hypnosis [24]. Additionally, there was a significant improvement in hair growth in 12 patients after an average of 5.5 hypnosis sessions. It is important to note that of these previously refractory hair loss cases, nine had total scalp hair regrowth [24]. However, on follow-up (anywhere from 4 months—4 years post-treatment), hypnosis was not found to prevent relapse. All patients who responded to hypnosis experienced at least minimal relapse, four of which returned to their pre-treatment status of alopecia [24].

Overall, these findings suggest that hypnotherapy has the potential to improve psychological wellness and alopecia, but without sustained benefits to hair loss. The limitations to this study include a small sample size, lack of control group, variability in the subjects’ concurrent AA interventions, and variability in the subjects’ number of hypnosis sessions [24]. A critical analysis of the methodological quality of this study, and the others included in this review is highlighted in Table 2.

In a consequent 2010 prospective cohort study, Willemsen et al. assessed hypnotherapy as a standalone treatment for alopecia with the use of a control group [33]. For 6 months, 20 patients received hypnosis, which consisted of a total of 10 hypnosis sessions scheduled bimonthly, and a daily self-hypnosis practice with an audio recording. Twenty-one patients served as control, continuing only on their usual alopecia treatment [33]. At baseline, and after 6 months of treatment, psychological well-being was measured with the SCL anxiety and depression subscales; quality of life was evaluated with the Skindex-17, and the 36-Item Short Form Survey (SF-36); and hair regrowth was assessed by visual examination and photography of the scalp [33]. The Skindex-17 examines the self-reported degree of functional and emotional impact of skin disease on patients with 17 items such as, “My skin condition makes me feel depressed” and “My skin condition affects my desire to be with people.” [34]. The SF-36 assesses 8 different components of health-related quality of life. These components are physical functioning, role limitations to due physical health, bodily pain, general health, vitality, social functioning, role limitations due to emotional disturbances, and mental health [35].

**Table 1:** Summary of psychological interventions studied in alopecia areata and scarring alopecia.

Study	Type of Hair Loss	Study Design	Sample Size	Intervention	Results
Gallo et al. (2017) [18]	Alopecia areata (moderate to severe)	Prospective cohort, with control	16 (8 intervention, 8 control)	Eight-week Mindfulness-Based Stress Reduction in addition to ongoing AA medical treatment.	In MBSR group: Significant improvement in AA-QLI subjective symptoms and relationship impacts (maintained at 6 months) Significant improvement in Brief Symptom Inventory on anxiety (maintained at 6 months), phobia, overall psychological distress, and global severity index.
Harrison and Stepanek (1991) [32]	Refractory alopecia (scarring and non-scarring alopecia)	Pilot study	5	10–12 forty-five-minute sessions of hypnotherapy over 3 months and recordings for home use.	All participants reported a feeling of well-being. Three patients: partial hair regrowth One patient: significant regrowth One patient: no change
Willemsen et al. (2006) [24]	12 with extensive alopecia areata, 9 with alopecia totalis or universalis	Prospective cohort	21	In person hypnosis sessions every 3 weeks along with hypnosis twice weekly from a recording at home.	Significant improvement in Symptom Check List, anxiety, and depression scales. Significant hair regrowth in 12 patients.
Willemsen et al. (2010) [33]	Alopecia areata, alopecia totalis and universalis, all for at least 3 months prior to enrollment	Prospective cohort, with control, nonrandomized	41 (20 intervention, 21 control)	10 hypnotherapy sessions performed bimonthly along with daily hypnotherapy from a recording at home. No concurrent hair loss treatments.	At 6 months, the hypnotherapy group reported significantly greater reductions in depression, anxiety, and symptom burden scores. Eight patients from hypnosis group experienced (non-significant) hair growth.
Willemsen et al. (2011) [19]	Alopecia areata	Prospective cohort	21	10 hypnotherapy sessions over 6-months and daily hypnosis recording at home.	At 6 months, the Symptom Check List, alexithymia, and Skindex-17 (dermatology related quality of life) scores all improved. This was maintained 6 months after treatment.
Teshima et al. (1991) [20]	Alopecia universalis (refractory)	Clinical trial, not blinded	11 (6 intervention, 5 control)	Psychoimmunotherapy: Six patients underwent 4 months of 30-min relaxation therapy in addition to oral prednisolone and later cyclosporine given to all participants.	In the psychoimmunotherapy group, five/six participants saw hair regrowth. There was also increases in scalp blood flow. In immunotherapy only group: one/five participants had regrowth.
Matzer et al. (2011) [36]	Alopecia areata	Cross sectional	45	Interview at baseline and 6 months evaluating coping strategies and disease burden.	Active and open coping strategies were associated with reduced disease burden in chronic AA.
Heapy et al. (2021) [21]	Alopecia areata	Case series	5 (data excluded from 1 due to missing data)	Eight-session mindfulness-based cognitive therapy (MBCT) intervention	Reduction in idiographic measures of social anxiety, with greater effects from baseline to follow-up than baseline to postintervention. Significant improvement in measures of well-being from baseline to follow-up in two participants who implemented MBCT exercises frequently between sessions.
Gorbatenko-Roth et al. (2021) [22]	Alopecia areata	Randomized, controlled pilot study	30 (20 intervention, 10 control)	AA-specific collocated behavioral health (CLBH) treatment, involving up to 2, 30-min sessions.	CLBH group reported better psychosocial functioning than control for most outcomes, although differences were nonsignificant CLBH was perceived as beneficial; 100% reported increased dermatology care satisfaction, 90% endorsed addressing psychosocial issues during dermatology visits.

**Table 2:** Critical analysis of methodological quality using the Jadad scale.

Item	Randomization /2 1 Point if Randomization Is Mentioned 1 Additional Point if the Method of Randomization Is Appropriate	Blinding /2 1 Point if Blinding Is Mentioned 1 Additional Point if the Method of Blinding Is Appropriate Deduct 1 Point if the Method of Blinding Is Inappropriate	An Account of All Patients /1 The Fate of All Patients in the Trial Is Known. If There Are No Data, the Reason Is Stated	Total Score /5 Max 5 Points
Gallo et al. (2017) [18]	0 No randomization	0 No mention of blinding	1 Results were reported with eight individuals in each group, implying all 16 participants completed the study.	1
Harrison and Stepanek (1991) [32]	0 No randomization/control group	0 No blinding	0 A total of 5/12 patients completed the study, but authors do not state why 7 did not complete the study.	0
Willemsen et al. (2006) [24]	0 No randomization/control group	0 No blinding	1 Only 21/28 patients completed the study, but authors state that 7 patients withdrew due to lack of motivation.	1
Willemsen et al. (2010) [33]	0 A nonrandomized controlled study protocol was selected for ethical reasons	0 No mention of blinding	1 Results were reported with 20 treatment patients and 21 control patients, implying all 41 participants completed the study.	1
Willemsen et al. (2011) [19]	0 No randomization	0 No blinding	1 A total of 24 patients were included in the study, and 3 patients dropped out (one due to lack of motivation, two due to failure to concentrate while listening to the audiotape for self-hypnosis).	1
Teshima et al. (1991) [20]	0 No randomization	0 No blinding	1 Results reported for 11/11 patients.	1
Matzer et al. (2011) [36]	0 No randomization	0 No blinding	1 Results reported for 45/45 patients. Two could not be contacted for 6-month follow up.	1
Heapy et al. (2021) [21]	0 No randomization	0 No blinding	1 Results reported for four/five patients (one excluded due to incomplete responses).	1
Gorbatenko-Roth et al. (2021) [22]	2 Randomized appropriately	0 No blinding	0 Participation and 1-month follow-up rates were 68% and 90%, respectively.	2

At the 6-month mark, those in the hypnosis group had significant improvements in anxiety and depression subscales, and in the mental component summary score of the SF-36 relative to control. With regards to alopecia outcomes, eight patients from the hypnosis group experienced hair growth by the end of treatment, but this was considered to be non-significant relative to baseline (<50% scalp growth) [33]. This study did not utilize objective image-based analysis with the technology that is available today but relied upon visual inspection and photography and was likely less robust of a methodology. Future studies of hypnosis should use more objective technology such as the phototrichogram based image analysis. Interestingly, the improved psychological outcomes reported were repeated by Willemsen et al. in a following study [19].

A 2011 prospective cohort study had 21 subjects participate in 10 one-hour sessions of hypnotherapy during a 6-month period, and similar to the previous study, subjects followed a self-hypnosis audio recording for about 20 min daily [19]. Here, the Toronto Alexithymia Scale-20 (TAS-20), the SCL, the SF-36, and the Skindex-17 were used as outcome measures and monitored at baseline, at the end of the 6-month treatment, and an additional 6-months after treatment [19]. No control group was utilized, and hair regrowth was not assessed. Relative to baseline, there was significant improvement in all of these questionnaires after the 6-month intervention period, and there was no significant change between at the additional 6 month observation period post-treatment [19]. This suggests that hypnosis was able to benefit psychological and quality of life parameters, and that these benefits were maintained after treatment [19].

Overall, the work of Harrison and Stepanek, and Willemsen et al. demonstrate the potential of psychological interventions, such as hypnosis, in the wellbeing of those experiencing hair loss [19,24,32,33]. The efficacy of hypnosis for hair loss itself will require further investigation with more robust hair analysis techniques.

### 3.4. Psychoimmunotherapy

Teshima et al. studied the effect of psychoimmunotherapy on alopecia universalis (AU) due to some evidence suggesting immunological and psychological abnormalities in AU [20]. These investigators set out to assess whether mental health improvements can modulate the impact of immunotherapy on the immune system by comparing immunotherapy alone to immunotherapy with psychotherapy on blood lymphocyte levels. To further understand associated pathophysiology and quality of life-related measures, the investigators examined ß-endorphin levels, blood flow, and temperature of the scalp, and self-portraits drawn by participants over the course of the study [20].

Eleven patients with refractory AU were included [20]. In each case, psychosocial disturbances including new employment, abortion, and a traffic accident were thought to be related to the cause of disease. All 11 patients received the immunotherapy which included oral prednisolone 5–10 mg/day taken alone for 2 months, then taken with the addition of cyclosporin (CYA) (2.5 mg/kg) for another 2 months [20]. Of these patients, six also received psychotherapy. The psychotherapy consisted of weekly 30-min sessions of relaxation and image therapy for 4 months. During image therapy, patients were asked to visualize hair cells receiving blood flow and nutrients, imagine head hairs growing, envision themselves as confident, and draw an image of themselves cured from AU [20].

There was a significant increase in the number of CD8-positive T cells in both treatment groups [20]. In psychoimmunotherapy patients who experienced hair regrowth, there was also an increase in the number of CD16-positive T cells, a decrease in the CD4/CD8 ratio, and a decrease in the total number of CD4-DR-positive T cells. Furthermore, the ß-endorphin levels, which may promote T cell activity, were found to significantly increase after relaxation and image therapy [20]. Overall, hair regrowth was seen in five/six cases of psychoimmunotherapy treatment, compared to only one/five cases where immunotherapy was used alone [20]. Interestingly, visualizing the scalp receiving blood flow and imagining it become warm was associated with increases in blood flow and temperature. Finally, with psychoimmunotherapy, self-portraits became more self-confident, such as the case of one patient who initially could not picture themself as cured but was later able to draw themself with hair regrowth and a joyous expression [20]. Overall, these findings suggest that complimenting immunotherapy with psychotherapy may improve the likelihood of AU recovery via immune modulation, increased blood flow, and improvements in self-image [20].

The findings are clinically relevant as patients engaged in some form of immunotherapy or psychological interventions are not likely using these interventions as monotherapy. Therefore, more studies that follow up on combination treatment therapy will be important.

### 3.5. Coping Strategies

Matzer et al. performed a study analyzing coping strategies in 45 patients with AA [36]. Coping strategies were assessed with the stress and coping process questionnaire (UBV) and with rating questions relating to the subjective stress and burden associated with AA. There was also a follow-up interview after 6 months to inquire about disease burden, and disease status to assess whether patients’ alopecia improved, worsened, or stayed the same [36].

Overall, for those experiencing their first episode of AA, passive coping strategies, such as waiting for hair regrowth, were associated with less subjective burden [36]. However, for those with chronic or relapsing AA, it was active and open coping strategies that were inversely associated with subjective burden [36]. These strategies included addressing psychological triggers, and openly discussing and displaying their hair loss [36].

Interestingly, the severity of AA and the disease status of AA (first episode, chronic, relapsing) were not found to influence the subjective burden of hair loss [36]. This suggests that the psychological and quality of life impacts of hair loss may be more related to a patient’s response to the hair loss, rather than to the hair loss itself [36].

Furthermore, with follow-up, disease improvement was associated with more advantageous coping strategies, which the authors defined as less aggressive responses to stressors and a belief in changeability of stressors [36]. Taken together, the work of Matzer et al. demonstrates the importance of psychological coping and emotional regulation in hair loss, and serves as a call for further investigation with larger samples and clinical trials [36].

## 4. Discussion

In the studies reviewed, the benefits of varying psychological interventions were often attributed to their potential modulation of the immune system and the stress response. While the underlying pathophysiology of alopecia is complex and multifactorial, it is well-established that there is an autoimmune component to AA [37]. Research suggests that there is a T-cell mediated attack on hair follicle antigens [38], and more specifically, that CD4 positive and CD8 positive T cells may play an important role in the pathogenesis [39].

In one study, McElwee et al. demonstrated hair growth in rats with AA after in-vivo depletion of CD4 positive T cells [40]. Similarly, the results of Teshima et al. demonstrate significant reductions in the in the total number of CD4 positive T cells in those who experienced hair growth from psychoimmunotherapy treatment [20]. Since these results were not seen in immunotherapy alone, perhaps the addition of psychotherapy has a distinct ability to modulate the immune system and promote hair growth [20]. In fact, emerging research suggests that various mind-body therapies may exert an anti-inflammatory effect through the modulation of immune response components which may be relevant to the inflammatory state in alopecia [41,42,43]. To illustrate, those with AA have been found to have significantly higher levels of inflammatory markers such as C-reactive protein (CRP) relative to control, and one systematic review suggests that mindfulness-based practices, such as MBSR, may decrease CRP [41,44]. Nevertheless, due to the complexity of the immune system, further research will be necessary to elucidate the impact of mindfulness and relaxation techniques on other immune markers associated with alopecia, such as type 2 and type 17 cytokines [43].

Of note, most patients with AA that are actively seeking treatment are likely to be on some form of immunotherapy. In light of baricitinib receiving FDA approval for the treatment of AA, most patients seeking treatment will likely consider a Janus kinase inhibitor (JAK) inhibitor as part of their therapy. Accordingly, more studies for the use of psychological interventions or hypnosis will be needed in combination with JAK inhibitor therapy for AA.

The stress response has also been found to affect hair follicles and interact with the immune system. In animal studies, psychological stress can lead to perifollicular inflammation, mast cell degranulation, and premature regression of the hair follicle [45]. Moreover, research suggests that the hypothalamic-pituitary-adrenal (HPA) axis is implicated in hair loss conditions [46]. Corticotropin releasing hormone, a key hormone in the HPA stress response, has been found to suppress hair shaft elongation and proliferation of human dermal papilla cells, induce the regression phase of the hair cycle, increase production of reactive oxygen species and catagen- (regression-) related molecules, and decrease anagen- (growth-) related molecules [46]. We hypothesize that psychological interventions such as mindfulness-based stress reduction, coping strategies, and the relaxation components of hypnotherapy and psychotherapy may be helpful in hair loss by offsetting the impact of the stress response on hair follicles. While future research will be needed to more closely analyze the HPA axis in these therapies, current literature demonstrates significant blood cortisol reduction with meditation in those with somatic illnesses [47].

With immunomodulatory mechanisms, hormonal pathways, and biological markers aside, all the psychological interventions reviewed have improved measures in quality of life and mental wellness in those experiencing hair loss. However, the improved psychosocial functioning measures reported by those receiving alopecia-specific CLBH were nonsignificant when compared to the control group [22]. Interestingly, psychological interventions may be helpful regardless of changes to hair loss [18,36]. As demonstrated with MBSR, the treatment group had significant increases in quality-of-life measures despite a lack of significant improvement in hair loss. As demonstrated by Matzer et al., coping strategies, but not AA severity or disease status, influenced the subjective burden of hair loss [18,36]. Given the immense psychological consequences associated with alopecia, we would like to use these results as a call for future clinical trials and integrative treatment approaches.

This review has several limitations. Overall, the number of studies exploring psychological interventions in conditions of alopecia are limited. Our research resulted in studies investigating only AA, alopecia totalis, alopecia universalis, and only two patient cases of scarring alopecia. We were not able to sufficiently review the role of psychological modalities on conditions of androgenic alopecia or broader studies of scarring forms of hair loss. Our review could also have had publication bias by not utilizing other databases such as Web of Science, PsycINFO, ScienceDirect, and Directory of Open Access Journals. Furthermore, in the existing literature that we have reviewed, small sample sizes, a lack of control and randomization, and variable outcome measures are factors that should be improved for future studies. A critical analysis of the articles included in this review has been completed using Jadad scoring and is summarized in Table 2. The Jadad score for the articles reviewed ranges between 0–2, indicating a need for higher quality trials with randomization, blinding, and accountability of all patients. Future studies should also utilize objective endpoints such as phototrichogram based measurements so that there are more quantitative results that can be tracked. Nevertheless, the findings are promising. The interventions included in this review may help pave a stable path for wellness and quality of life in the unpredictable course of hair loss.

## 5. Conclusions

The results of this review suggest that mindfulness practices, hypnotherapy, psychotherapy, and coping strategies may have early evidence to improve measures of quality of life and mental health in patients with various forms of alopecia. Though the data and measurements methodologies are limited, improvements in hair growth have also been demonstrated with the use psychotherapy and hypnotherapy, where its significance is inconclusive. Larger and more rigorous studies are necessary to establish the efficacy and optimal administration of psychological interventions in conditions of hair loss.

## Data Availability

No primary data was generated in this review.
